# Preparation and Properties of Layered SiC-Graphene Composites for EDM

**DOI:** 10.3390/ma14112916

**Published:** 2021-05-28

**Authors:** Ondrej Hanzel, Zoltán Lenčéš, Peter Tatarko, Richard Sedlák, Ivo Dlouhý, Ján Dusza, Pavol Šajgalík

**Affiliations:** 1 Institute of Inorganic Chemistry, Slovak Academy of Sciences, Dúbravská Cesta 9, 845 36 Bratislava, Slovakia; zoltan.lences@savba.sk (Z.L.); peter.tatarko@savba.sk (P.T.); pavol.sajgalik@savba.sk (P.Š.); 2 Institute of Materials Research, Slovak Academy of Sciences, Watsonova 47, 040 01 Košice, Slovakia; rsedlak@saske.sk (R.S.); jdusza@saske.sk (J.D.); 3 Institute of Physics of Materials, Czech Academy of Sciences, Žižkova 513/22, 616 00 Brno, Czech Republic; idlouhy@ipm.cz

**Keywords:** SiC, graphene, layered composites, electrical conductivity, scratch tests

## Abstract

Three and five-layered silicon carbide-based composites containing 0, 5, and 15 wt.% of graphene nanoplatelets (GNPs) were prepared with the aim to obtain a sufficiently high electrical conductivity in the surface layer suitable for electric discharge machining (EDM). The layer sequence in the asymmetric three-layered composites was SiC/SiC-5GNPs/SiC-15GNPs, while in the symmetric five-layered composite, the order of layers was SiC-15GNPs/SiC-5GNPs/SiC/SiC-5GNPs/SiC-15GNPs. The layered samples were prepared by rapid hot-pressing (RHP) applying various pressures, and it was shown that for the preparation of dense 3- or 5-layered SiC/GNPs composites, at least 30 MPa of the applied load was required during sintering. The electrical conductivity of 3-layered and 5-layered composites increased significantly with increasing sintering pressure when measured on the SiC surface layer containing 15 wt.% of GNPs. The increasing GNPs content had a positive influence on the electrical conductivity of individual layers, while their instrumented hardness and elastic modulus decreased. The scratch tests confirmed that the materials consisted of well-defined layers with straight interfaces without any delamination, which suggests good adhesion between the individual layers.

## 1. Introduction

The costly diamond-tool machining of hard ceramic materials, such as SiC, TiC, and others, remarkably increases the price of products. Therefore, there is a tendency to apply cheap electrical discharge machining (EDM) for cutting the required ceramic parts from bulk materials. EDM is a contactless method of machining various materials by the series of sparks generated between the work piece electrode and tool electrode immersed in dielectric fluid. It is an effective method for machining very hard and/or brittle materials like ceramics. The majority of ceramic materials are insulators, therefore not suitable for EDM, as the electrical conductivity of the work piece should not be lower than 1 S/m [1]. However, our previous experiences on the electrical-discharge machining of Al_2_O_3_-CNTs and SiC-GNPs composites showed that for effective EDM the electrical conductivity of machined materials should be at least 500 S/m [2,3]. There are two main approaches to increase the electrical conductivity of non- or less-conducting ceramic materials to be suitable for EDM: (i) addition of electrically conductive secondary phase, e.g., carbon nanotubes (CNTs), graphene, metals, etc., or (ii) creating a conductive layer on the surface of work piece which allows using the assisting electrode method of EDM (ASEDM) [4]. The conductive surface layer helps to trigger the initial spark between the work piece and tool electrode. During the ASEDM process, a layer of pyrolytic carbon is formed from the dielectric liquid medium (e.g., kerosene), which enables the continuous EDM process [5].

There have been many studies reporting on the effect of carbon nanostructures (CNTs, GNPs, carbon fibers) addition on the electrical and thermal conductivity of ceramic composites [2,6,7,8,9,10,11,12,13,14]. Clear enhancement of electrical conductivity of various ceramics by incorporation of graphene was confirmed by many authors, e.g., Sedlák et al. improved electrical conductivity of the reference B_4_C material with 4 wt.% of GNPs (σ = 70 S/m in the parallel direction to graphene layers and σ = 40 S/m in the perpendicular direction) by adding 6 wt.% of GNPs up to value of 1526 S/m in parallel directions to the graphene layers and 872 S/m in perpendicular directions [7]. Tan et al. have made even more significant improvements of the electrical conductivity of B_4_C from a value of around 100 S/m up to 3250 and 5000 S/m in perpendicular and parallel directions to graphene layers by incorporation of 4 vol.% of GNPs into the matrix [12]. Centeno et al. increased the electrical conductivity of alumina, a material with high resistivity, by adding a very low graphene loading (0.22 wt.%) up to eight orders of magnitude in comparison to the monolithic alumina [9]. Ramirez et al. dispersed a relatively high amount of GNPs (25 vol.%) in silicon nitride matrix and reached maximum conductivity of 4000 S/m, and also showed remarkable anisotropic effect due to the preferential orientation of graphene during spark plasma sintering (SPS) [8].

In the case of in-plane thermal conductivity, a certain improvement is usually observed with an increasing amount of graphene nanoplatelets, while the cross-plane thermal conductivity decreases [2,13,14]. This is a consequence of a larger interfacial area in the direction perpendicular to graphene layers, which is accompanied by higher phonon scattering on boundaries, pores, defects, etc., and leads to a more pronounced reduction of thermal conductivity.

The electrical and thermal properties of ceramics–graphene composites strongly depend not only on the alignment of GNPs [2,6,13,14,15], but also on the homogeneous distribution of GNPs in ceramic matrices [2,6,13], the content of defects on the surface of GNPs, and the overall porosity of materials [16]. Moreover, the microstructure (grain size, thickness, and crystallinity of grain boundaries) [17,18,19], the concentration of impurity atoms including lattice oxygen in matrix grains [17,19,20], and the conditions of additional heat treatment after sintering [6,21] also affect the functional properties of ceramic–graphene composites. The enhancement of electrical conductivity of ceramic–graphene composites in both in-plane and cross-plane directions is more straightforward than in the case of thermal conductivity, as was confirmed by many studies [2,6,7,8,9,11,12,22]. Electrical conductivity usually increases with an increasing amount of graphene nanoplatelets in both directions. However, higher in-plane electrical conductivity was observed due to the intrinsic anisotropy in the electrical conductivity of GNPs. In addition, the alignment of GNPs in the composite matrix also leads to certain anisotropy, as more conductive paths are formed in the direction parallel to GNPs.

The hardness of ceramic composites usually decreases with increasing GNPs as a consequence of the incorporation of a much softer phase into the ceramic matrix [15,23,24,25,26]. One of the ways to overcome this problem and at the same time to allow EDM of ceramics–graphene composites is to develop layered materials. Such materials would consist of a conductive outer layer (serving as an assisting electrode) with a higher amount of graphene, while the core would be formed of monolithic ceramics or ceramics with a lower content of GNPs. Liu et al. showed that the incorporation of a small amount of GNPs (up to 3 vol.%) in the TiC matrix did not deteriorate the hardness but even led to improved hardness when compared to pure TiC [27]. Although the concept of layered ceramic materials was proposed more than 30 years ago [28], to date, there have been only few published papers related to ceramic/graphene layered composites [29,30,31,32,33]. For example, An et al. fabricated bioinspired graphene/ZrB_2_ ceramic materials with hierarchically ordered architectures, which exhibited a unique combination of high strength (522 MPa) and toughness (9.5 MPa.m^1/2^), especially composite toughness was improved by the combination of various toughening mechanisms, including the sliding of graphene nanosheets, crack deflection, graphene crack pulling out and bridging, and crack branching [30]. Rincón et al. prepared layered yttria-stabilized zirconia (YSZ), with or without graphene, by colloidal processing followed by SPS, and demonstrated that the addition of GO-enriched layer into YSZ laminates resulted in an increase of fracture toughness [31]. Balazsi et al. prepared porous 3-, 5-, and 7-layered Si_3_N_4_-graphene composites by stacking alternate layers with 5 and 30 wt.% of GNPs followed by hot isostatic pressing (HIP) [32,33]. The first intention for the design of these layered materials was to improve the strength and toughness of ceramics. However, this material design can also be used for the enhancement of functional properties in the preferred direction for applications that include, e.g., thermal or electromagnetic interference shields or EDM.

The main goal of this study was the preparation of dense 5-layered SiC-graphene materials with stepwise increase of electrical conductivity from the middle part towards the surface of composite, in order to obtain a sufficiently high electrical conductivity (σ ≥ 500 S/m) in the outer layer for effective assisting electrode EDM of these materials from both sides. The materials were designed to contain different amounts of GNPs in the individual layers (from 0 to 15 wt.%) with the following layer sequence: SiC-15GNPs/SiC-5GNPs/SiC/SiC-5GNPs/SiC-15GNPs. The materials processing consisted of sequential uniaxial pressing of the powders with different amounts of GNPs, followed by electric-field assisted sintering. In order to optimize the processing and verify a sufficient adherence of the individual layers, 3-layered composites with layer sequence SiC/SiC-5GNPs/SiC-15GNPs were also prepared. The density, Raman spectra, microstructures, hardness, elastic modulus, and scratch tests across the 3-layered composites were investigated. In addition, the electrical conductivity and thermal diffusivity of both the 3-layered and 5-layered composites were also studied.

## 2. Materials and Methods

### 2.1. Powder Preparation

For the preparation of reference and composite powders SiC (grade HSC-059, β-SiC, d_50_ = 0.55 μm, Superior Graphite, Chicago, IL, USA), Y_2_O_3_ (purity > 99.99%, HC Starck, Goslar, DE), α-Al_2_O_3_ (grade TM-DAR, purity > 99.99%, particle size 100 nm, Taimei Chemicals Ltd., Tokyo, Japan) and graphene nanoplatelets (thickness < 3 nm, purity 99%, lateral size 1–2 μm, Cheap Tubes Inc., Grafton, VT, USA) were used. The layered materials were prepared using three different powder compositions in order to maintain the same compositions as that of reference (non-layered) composites [2]. The reference powder consisted of SiC:Y_2_O_3_:Al_2_O_3_ in a proportion of 93:5:2 wt.%. The other two powder mixtures additionally contained 5 wt.% and 15 wt.% GNPs, respectively. The GNPs were ultrasonically agitated for 60 min by an ultrasound probe (Sonopuls HD 3200, Bandelin Electronic GmbH, Berlin, Germany) prior to the addition to the SiC–Y_2_O_3_–Al_2_O_3_ matrix powder. The powder mixtures were ball milled in distilled water in a plastic jar with SiC balls on rollers for 24 h. The suspension was sprayed into the liquid nitrogen, and subsequently, the frozen powders were freeze-dried in order to remove the ice by sublimation. This procedure is crucial for ensuring the homogeneous distribution of GNPs in SiC matrix. The powder mixtures were dried at 80 °C for 12 h, afterwards sieved through a 300 μm microscreen.

### 2.2. Preparation of Layered and Reference Composites

**Figure 1:**
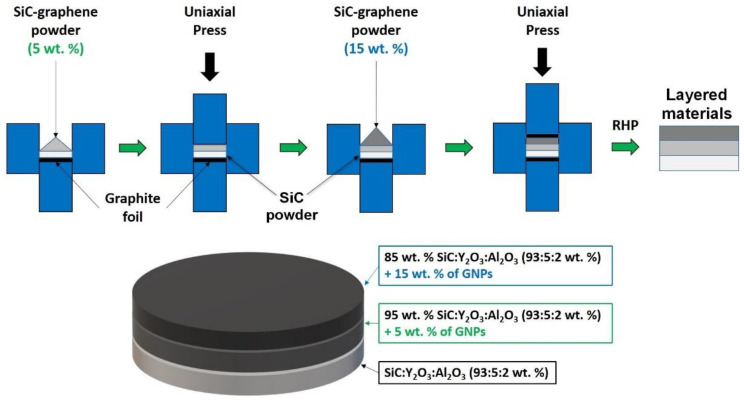
Schematic illustration of the preparation of layered materials.

The layered materials consisting of 3 or 5 layers were prepared directly in a graphite die (*ϕ* 20 mm) by precise weighing, pre-pressing of each layer (30 MPa), and final uniaxial pressing of pellets using 30 MPa load (Figure 1). In the asymmetric 3-layered samples, the layer sequence was 0-5-15% GNPs, while in the symmetric 5-layered samples, it was 15-5-0-5-15% GNPs. The thickness of layers was controlled by the weight of composite powders. The sintering of samples was performed in rapid hot press (DSP 507, Dr. Fritsch GmbH., Fellbach, Germany) at 1800 °C for 5 min applying various uniaxial pressures (minimal contact pressure ~7 MPa, 30 MPa, 40 MPa, and 50 MPa), under vacuum. Contrary to the fast heating rate (100 °C/min), a relatively slow cooling rate (20 °C/min) was used during sintering to minimize the residual stresses in the sintered layered samples. After sintering, a surface layer with a thickness of approximately 1 mm was ground in order to remove the carbon contaminated layer. The layered samples are denoted as C*n-x*, where *n* is the number of layers and *x* refers to the applied sintering pressure. For example, C_3-40_ refers to the 3-layered composites sintered under 40 MPa applied load. For the sake of comparison, the pellet-shaped non-layered samples with a diameter of 20 mm and a thickness of 4 mm were prepared from the individual compositions (SiC, SiC-5% GNP, and SiC-15% GNP) in our earlier work [2] by rapid hot-pressing at 1800 °C for 5 min applying a uniaxial pressure of 50 MPa.

### 2.3. Characterizations of the Layered Composites

Densities of the prepared composites were measured by the Archimedes method in distilled water. The theoretical density of each layer (*ρ_i_*) was calculated by the rule of mixtures using a density of 3.2 g·cm^−3^ for SiC, 4.0 g·cm^−3^ for Al_2_O_3_, 5.0 g·cm^−3^ for Y_2_O_3_, and 2.2 g·cm^−3^ for GNPs, respectively. The theoretical densities of 3- and 5-layered composites were calculated by combining the theoretical densities of individual layers (*ρ_i_*) and their layer thickness (*x_i_*) using the equation [34]:

(1) $$\rho =\overset{3-5}{\underset{i=1}{\sum }}\frac{x_{i}}{X_{T}}\rho_{i}$$

where *X_T_* is the total thickness of layered composites.

Scheme 40. HV (Carl Zeiss, Munich, Germany) was used for the microstructural analysis of composites. The sintered layered samples were polished and plasma etched using CF_4_ + 10% O_2_ gas mixture (Diener electronic, Plasma-Surface-Technology, Stuttgart, Germany). The mean grain size in the individual layers was estimated by the linear intercept method (Lince software 2.4.2β, TU Darmstadt, Germany). An optical digital microscope VHX-1000 (Keyence, Mechelen, Belgium) was used for the precise measurements of the layer thicknesses and verification of the positions of individual indents.

Raman spectra were recorded on the cross-sections of 3-layered composites, in the center of each layer using a DXR Raman Microscope (Thermo Fisher Scientific Inc., Boston, MA, USA) equipped with an Ar laser (λ = 532 nm). For each layer, at least five Raman spectra were recorded.

The instrumented hardness and indentation elastic modulus of composites were measured using an instrumented hardness machine Zwick ZHU/Z2.5 (Zwick/Roell, Haan, Germany) equipped with a Vickers indenter and applying a maximum load of 9.81 N. The load and penetration depth were continuously recorded during each test. The indentation elastic modulus was obtained from the unloading branch of the curves. Cross-sections of specimens for scratch tests were prepared by routine ceramographic procedure, they were cut, ground, and polished to a 1 µm finish. The scratch tests were conducted with the Bruker UMT-2 tool using a Rockwell tip to determine the friction and wear behavior of samples in dry sliding. For each specimen, 3 scratches of 5 mm length were performed through all layers with gradually increased applied load up to 20 N. Testing was carried out in air at room temperature.

The electrical resistivity of the sintered samples was determined by using a standard four-point Van der Pauw method, Loresta-AX MCPT370 (NH Instruments, Willich, Germany) with a linear configuration of probe tips. Before the analysis, the surfaces of the specimens were polished to a mirror finish. In the case of asymmetric 3-layered composites, the electrical resistivity was measured from both sides (from the SiC and S15GNP layers, respectively). In the case of symmetric 5-layered composites, the electrical resistivity was also measured from both sides, although the outer layers were identical (S15GNP layer).

The thermal diffusivities were measured using the laser flash analyzer LFA 1000 (Linseis Messgeraete, Selb, Germany) in the temperature range from room temperature to 400 °C in a vacuum. Prior to the measurements plan, parallel surfaces were prepared by a diamond wheel profile grinding machine JE 525 P (K. JUNG, Hamburg, Germany). A thin graphite layer was sprayed onto both sides of the dried samples to hinder any reflection of the laser beam. At least three measurements were done at each measuring temperature. The thermal diffusivities of 3-layered composites were measured from both sides, i.e., from SiC and S15GNP layers, respectively.

## 3. Results and Discussion

### 3.1. Densities and Microstructure of Layered Composites

The relative densities (RD) of 3-layered and 5-layered composites are summarized in Table 1. Except for the samples sintered under a minimum contact pressure (C3-7 and C5-7), the other samples were almost fully dense with relative densities higher than 97%. There was only a moderate increase of density (from 97.5 to 98.8%) with the increasing hot-pressing pressure from 30 MPa to 50 MPa. The results showed that for the preparation of dense 3- or 5-layered SiC/GNP composites, at least 30 MPa of the applied load was required during sintering.

The thicknesses of individual layers in 3-layered and 5-layered composites are given in Table 1. As expected, the layer thicknesses slightly decreased with the increasing sintering pressure, suggesting the improved densification with the increasing pressure. This was also confirmed by the density measurements.

**Figure 2:**
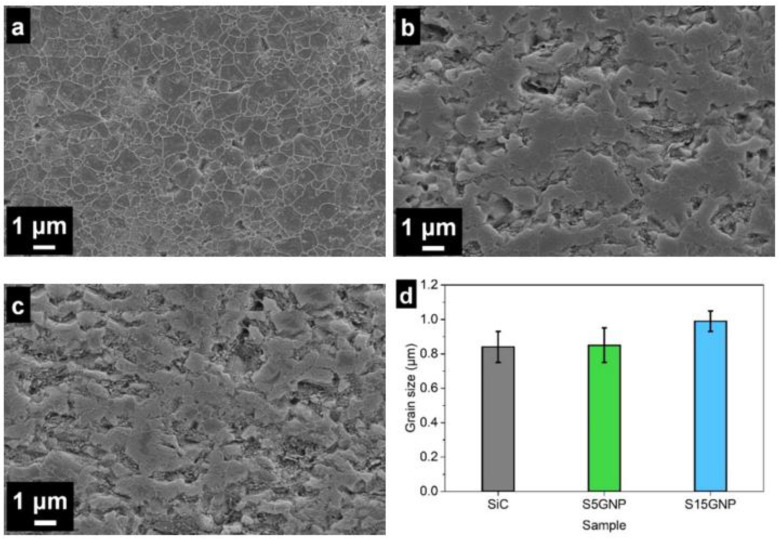
Plasma etched surfaces of individual layers in 3-layered composites sintered under 50 MPa pressure (C3-50) and grain size distribution of SiC. (**a**) 0% GNPs, (**b**) 5% GNPs, (**c**) 15% GNPs, (**d**) grain size of individual layers.

The SEM images of polished and plasma etched surfaces of individual SiC-based layers in 3-layered composite are shown in Figure 2a–c, while the grain size distribution is given in Figure 2d, respectively. It seems from Figure 2a–c that the microstructure became more porous with the increasing graphene nanoplatelet content. However, the measured densities were higher than 98% of TD. Therefore, it is assumed that the majority of surface pores were created by the removal of GNPs and pull out of SiC grains during grinding and polishing. Kovalčíková et al. observed a similar “porous” structure in the TiB_2_–SiC–GNP composites after the ceramographic procedure, but they proved the samples were fully dense using a focused ion beam milling technique [25]. The microstructure observations of 3-layered composites showed that in all layers, equiaxed grains of β–SiC were observed. The average grain size of SiC was in the range from 0.84 ± 0.09 μm to 0.99 ± 0.06 μm in all layers (Figure 2d). The layer with 15 wt.% of GNPs had a slightly larger grain size (within the standard deviation) which probably could be attributed to local overheating during electrically assisted sintering as a consequence of the higher electrical conductivity of this layer. However, we can claim that the presence of graphene nanoplatelets in the SiC matrix had no significant influence on the grain size of SiC.

### 3.2. Raman Spectra of Individual Layers

**Figure 3:**
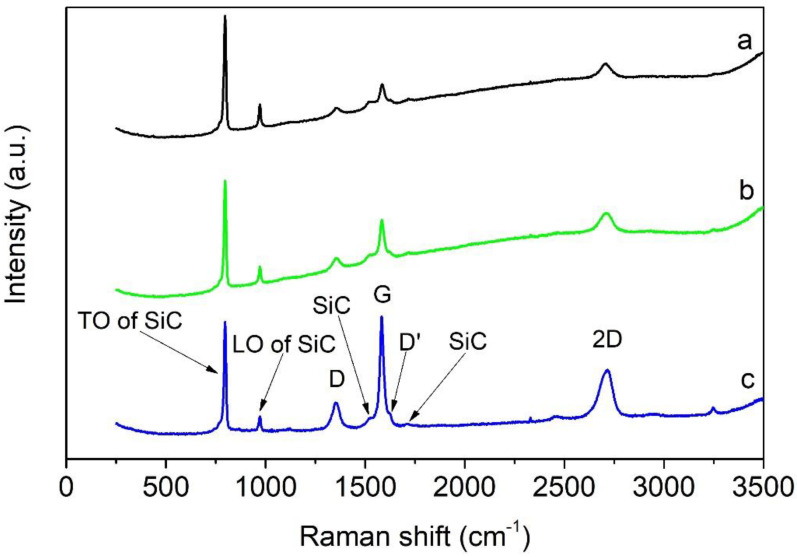
Raman spectra of individual layers in 3-layered composites: (**a**) SiC layer, (**b**) S5GNP layer, (**c**) S15GNP layer.

The Raman spectra of the individual layers in the 3-layered composites are shown in Figure 3. Typical features for SiC and graphene phases were clearly distinguished in each layer. The two characteristic peaks for cubic 3C-SiC are so-called transverse optical (TO) and longitudinal optical (LO) modes at 795 and 970 cm^−1^, respectively [35]. The weak peaks in the range of 1450–1700 cm^−1^ could be attributed to the second-order scattering of SiC [36,37]. The intensity ratio of TO and LO bands in the composite indicated that β–SiC was a major phase, and only traces of α–SiC were present [10,38]. The presence of graphene in all layers was confirmed based on the three main characteristic bands for graphene structures. The D-band at 1350 cm^−1^ indicates the breaks in translational symmetry of the hexagonal lattice, the G-band at 1585 cm^−1^ is related to C-C tangential vibrational mode of graphene-like surfaces, and the 2D-band at 2706 cm^−1^ originates from the double resonance process [39]. The intensities of D, G, and 2D bands increased with the increasing amount of graphene nanoplatelets in the SiC matrix. Moreover, the D’ band at 1620 cm^−1^, which originates from a double resonance process involving q-2k phonons close to the Brillouin zone center [40], was also visible. Interestingly, the characteristic graphene peaks were also observed in the SiC layer without the addition of GNPs. The in situ formation of graphene in SiC ceramics sintered by electric-field assisted technique has already been explained in the work of Miranzo et al. [10] and also in our earlier work [41].

### 3.3. Hardness, Indentation Elastic Moduli and Scratch Tests of Layered Composites

**Figure 4:**
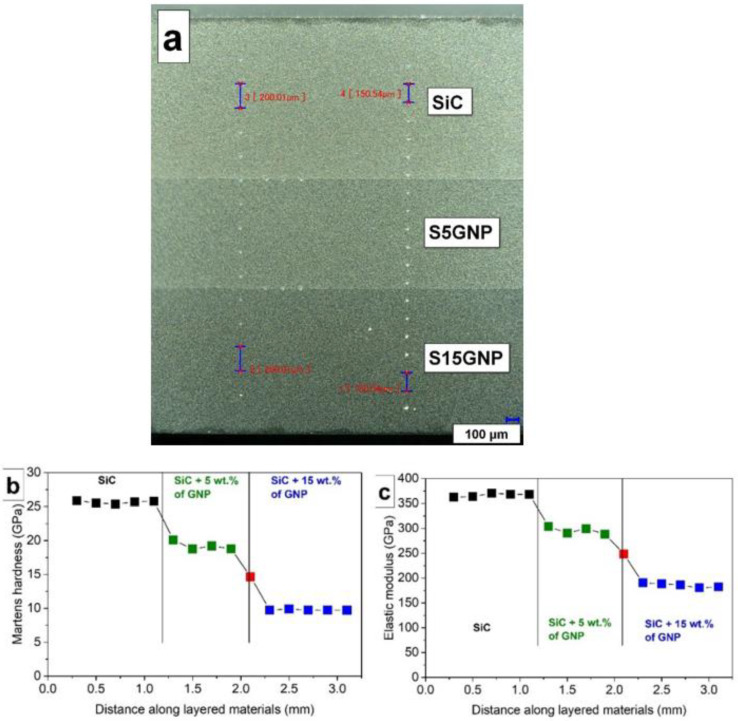
Optical micrograph of the cross-section of 3-layered composites–C3-50 (**a**), instrumented hardness (**b**), and indentation elastic moduli (**c**) of the layered composites measured on the cross-section.

The hardness and elastic modulus of 3-layered composites were investigated along the gradient direction, with 150 μm and 200 μm distances between the indents, respectively (Figure 4a). The optical micrograph also shows that the layers were well defined, strongly bonded with straight interfaces. No delamination of the layers was observed during cutting and grinding of the samples. The instrumented hardness varied with the position of indents in the 3-layered composites and abruptly changed at the layer interfaces (Figure 4b). The hardness decreased with the increasing GNPs content in the layers from 25.6 ± 0.4 GPa for the SiC layer to 18.9 ± 0.7 GPa for the S5GNP layer and 10.0 ± 0.5 GPa for the S15 GNP layer. This was caused by the increasing amount of a softer phase, i.e., GNPs. A similar decrease of hardness with an increasing amount of GNPs in the SiC matrix was also observed by Llorente et al. [23] or Sedlák et al. [42]. For example, Llorente et al. reported a hardness of 9.8 ± 1.1 GPa for the composites with similar composition, i.e., SiC:Y_2_O_3_:Al_2_O_3_ (93:5:2 wt.%) containing 14.6 wt.% of GNPs, which is in good agreement with our results. It can also be noticed in Figure 4b that the hardness measured at the interface between two layers (red square, *HV* = 14.7 GPa) was between the hardness values of the individual layers. The indentation elastic moduli followed the same trend like hardness, as it decreased with the increasing GNP content in the layers (Figure 4c). The indentation elastic modulus decreased from 363.0 ± 5.5 GPa for the SiC layer to 290.7 ± 6.7 GPa for the S5GNP layer and 187.2 ± 5.5 GPa for the S15GNP layer, respectively. Despite the high elastic modulus of graphene monolayer (1 TPa) it is well known that if graphene nanoplatelets or graphene oxide is incorporated in a ceramic matrix, the elastic modulus significantly decreases with an increasing amount of GNPs or GO [43].

**Figure 5:**
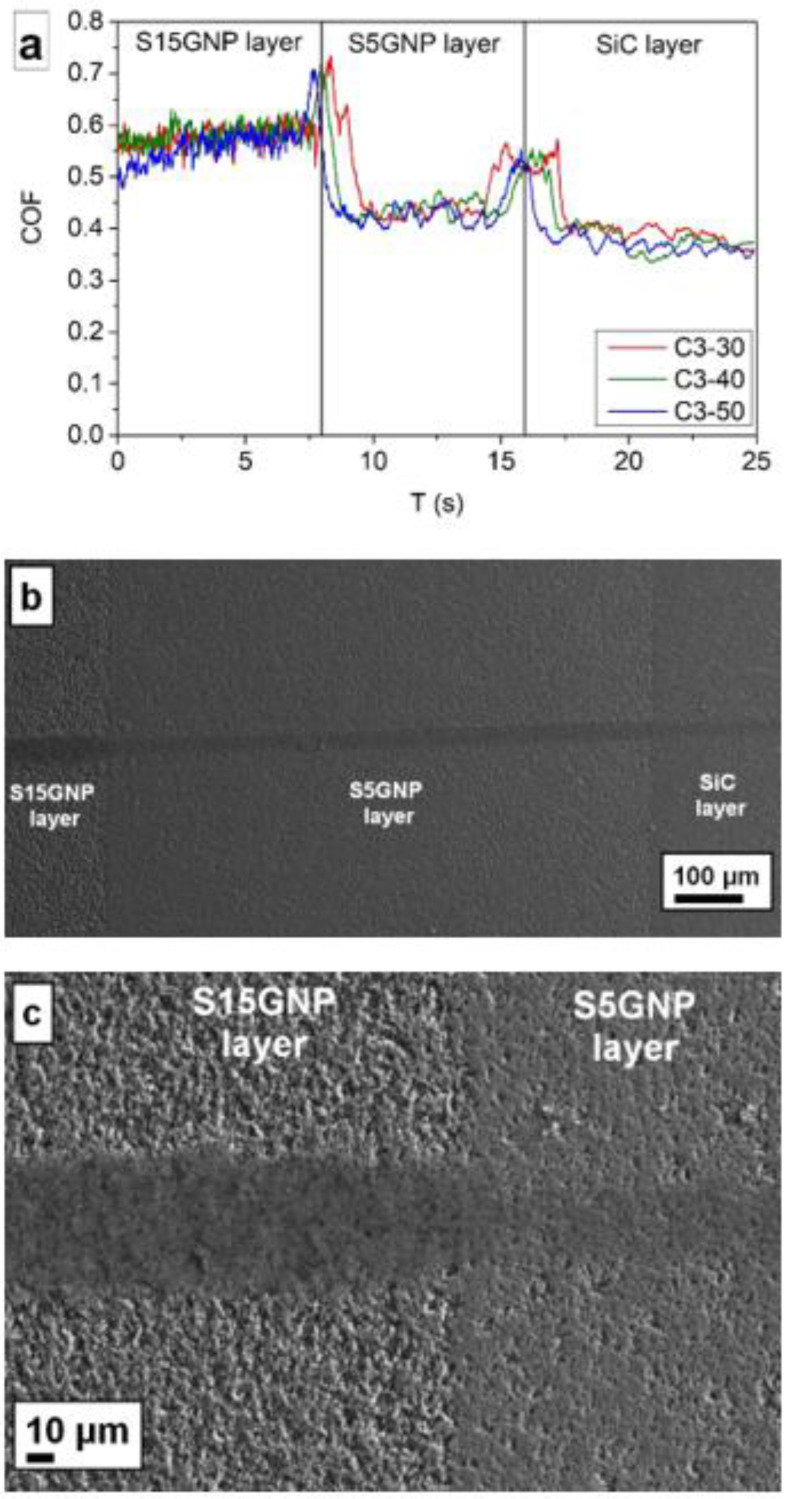
Scratch tests of 3-layered composites C3-x (where x = 30, 40, 50), (**a**) coefficient of friction of 3-layered composites measured by scratch tests (**b**) macrostructure with visible scratch after test and (**c**) detail of the scratch between two layers in C3-50 sample.

Scratch tests of the C3-x samples, where x = 30, 40, 50, were performed on the cross-sections of layered composites (Figure 5). No significant differences between the samples sintered at different pressures (30, 40, and 50 MPa) were observed. In each sample, three regions with different coefficient of friction (COF) were clearly distinguished, which corresponded to the individual layers. The SiC layer with 15 wt.% GNPs had the coefficient of friction in the range from 0.5 to 0.62, then the coefficient of friction decreased to the level of 0.40–0.43, followed by a very moderate decrease for the SiC layer (COF ~ 0.38). The interfaces between the layers are clearly recognizable by the abrupt increase of the coefficient of friction. The present results indicated that no lubrication effect of GNPs was observed. Contrary, the removed GNPs during grinding and polishing of samples made the surface rougher and the pull-out of SiC grains during scratch test became more significant with increasing GNPs content. This explains the higher COF for the layers with a higher amount of GNPs. Similarly, a significantly lower hardness of the layers with GNPs could negatively influence the COF. However, the most valuable information obtained was that no delamination of the layers was observed during the scratch tests, which suggests a good adhesion of the individual layers (Figure 5a,b).

### 3.4. Thermal Diffusivities of Layered Materials

**Figure 6:**
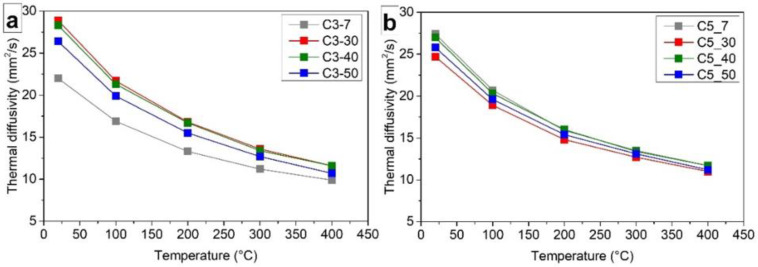
Thermal diffusivities of (**a**) 3-layered and (**b**) 5-layered SiC/GNP composites sintered under different pressures (7, 30, 40, and 50 MPa).

The thermal diffusivities of 3-layered and 5-layered composites sintered under different pressures (7, 30, 40, and 50 MPa) were investigated in a temperature range 25–400 °C (Figure 6) and was measured in the direction perpendicular to the layer sequences, i.e., perpendicular to the aligned GNPs in the S5GNP and S15GNP layers. In the case of 3-layered samples, only the thermal diffusivities measured from the S15GNP side are reported in Figure 6a, as there were no differences in thermal diffusivity values measured either from the SiC side, or S15GNP side, respectively. The thermal diffusivity of C3-7 layered sample sintered by minimum applied pressure was around 22.2 ± 0.2 mm^2^/s at RT, mainly due to the lower relative density. The thermal diffusivities of the samples sintered under higher pressures, which also exhibited higher densities, were in the range from 26.2 ± 0.1 to 29.2 ± 0.1 mm^2^/s.

The thermal diffusivities of 5-layered composites are shown in Figure 6b. The values varied from 24.7 ± 0.2 to 27.4 ± 0.2 mm^2^/s, i.e., the thermal diffusivities were slightly lower compared to those measured for the 3-layered composites at RT (from 26.2 ± 0.1 to 29.2 ± 0.1 mm^2^/s). This can be attributed to the higher number of defects at the SiC-GNP interfaces and at the boundaries between the layers, as there were two more layers with 5 and 15 wt.% of GNPs compared to the 3-layered composites. These defects caused phonon scattering and decreased the effective path for heat transport, which resulted in the decrease of thermal diffusivity. Except for the layered composites, Table 1 also summarizes the thermal diffusivities of reference non-layered samples, i.e. SiCref, S5GNP, and S15GNP. It is important to note that the reference non-layered samples were also dense, the relative density was higher than 97% even for the sample containing 15 wt.% of GNPs [2]. The comparison of thermal diffusivities of non-layered materials and layered composites sintered under 50 MPa shows that the thermal diffusivity of 3-layered sample is close to the value obtained for the reference S15GNP sample (Table 1). The thermal diffusivities of the layered samples C3-50 and C5-50 were slightly higher than that of the reference S15GNP sample, due to the higher thermal conductivity of SiC and S5GNP layers. However, the thermal diffusivity of the C3-50 layered sample was still lower than that of the reference S5GNP bulk sample, due to the additional boundaries on the layer interfaces, which acted as phonon scattering points and decreased the thermal diffusivity.

### 3.5. Electrical Conductivity of Layered Materials

**Figure 7:**
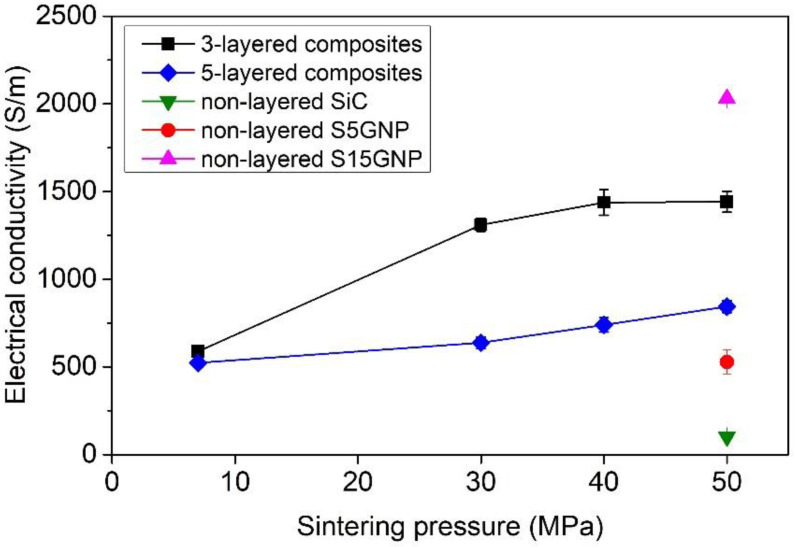
Influence of applied pressure during sintering on the electrical conductivity of 3- and 5-layered composites from S15GNP layer side and comparison with non-layered composites SiC, SiC with 5 wt.% and SiC with 15 wt.% of GNPs.

The electrical conductivities (σ) of 3-layered and 5-layered composites were also measured from both sides (i.e., SiC side and S15GNP side) and were investigated as a function of applied pressure during sintering (Figure 7). Unlike the thermal diffusivity, the electrical conductivity values strongly depend on the measurement direction, because the electric current flows mainly through surface layer and only partially through the bulk when Van der Pauw method is used Therefore, the electrical conductivities measured on the S15GNP side were much higher than the conductivities measured from the SiC layer side (10 < σ < 122 S/m, not shown in Figure 7) due to the conductive channels formed by graphene networks. In the SiC layer, only in-situ formed graphene domains were present. The presence of in situ formed graphene domains in the SiC layer was not sufficient to significantly improve the electrical conductivity. The electrical conductivity increased in both cases with the increasing sintering pressure, i.e., with the increasing density of the samples. However, the increase of electrical conductivity was more significant for the samples measured from the S15GNP side. The electrical conductivity of the C3-7 sample with a minimum contact pressure was 588 S/m, while it increased with the increasing pressure/density up to 1442 S/m for the C3-50 sample. Sample C3-7 showed the lowest electrical conductivity among all of the investigated materials due to its higher porosity. The most remarkable difference in electrical conductivity was between C3-7 and C3-30 samples (588 S/m vs. 1310 S/m) due to the large difference in their relative densities. A slight increase of electrical conductivity with the increasing sintering pressure (40 MPa or 50 MPa) followed the moderate increase of relative densities of these samples. In the case of measurements from the SiC side, the electrical conductivity increased linearly from 12 S/m for the C3-7 sample up to 122 S/m for the C3-50 sample. The present results showed that the electrical conductivity of the C3-50 sample is about 25% lower than the value obtained for the reference non-layered SiC material containing 15 wt.% of GNPs, and approximately three times higher than the electrical conductivity of reference non-layered material containing 5 wt.% of GNPs (Table 1).

Since the 5-layered composites were symmetric, there was no influence of the side of the measurements on the electrical conductivity, and the results were within the standard deviation of data. The electrical conductivity linearly increased with the increasing applied pressure (and density) from 513 S/m for sample C5-7 up to 869 S/m for sample C5-50. Although the electrical conductivities of 5-layered composites were measured on the S15GNP surface layer, which exhibited good conductivity (σ ~2000 S/m), the average value for the C5-50 sample was nearly half of the value measured for the reference non-layered material containing 15 wt.% of GNPs (Table 1). The lower electrical conductivity of the 5-layered composites was caused by the presence of the layers with lower intrinsic electrical conductivities (S5GNP and SiC). The present results clearly demonstrated that the preparation of 5-layered SiC-based composite with distinct layers containing different amounts of GNPs was successful. The individual layers strongly adhered to each other and defect-free interfaces were obtained between the layers. The final materials exhibited a sufficient electrical conductivity (600–870 S/m) for EDM machining, while consisting of a core with gradually improved mechanical properties, such as hardness and indentation modulus.

## 4. Conclusions

Asymmetric 3-layered and symmetric 5-layered composites were prepared by rapid hot pressing from SiC powders with Y_2_O_3_-Al_2_O_3_ sintering additives and different amounts of GNPs (0, 5, and 15 wt.%) as the electrically conductive phase. The rapid hot-pressing was carried out at 1800 °C for 5 min under different pressures (7, 30, 40, and 50 MPa). The following conclusions are drawn:

1. Stacking of freeze-dried powders with different amounts of homogeneously distributed graphene nanoplatelets among SiC particles, followed by relatively fast electric-field assisted sintering is a simple and effective method for the preparation of layered composites.
2. The relative density of sintered samples was higher than 97% when at least 30 MPa pressure was applied during sintering, even though layered composites contain a relatively high content of GNPs in specific layers (15 wt.%). Rapid hot pressing is an effective sintering method for the preparation of dense ceramic/graphene composites.
3. The GNPs content had an influence on the thermal and electrical properties of layered samples. The thermal diffusivities of 3- and 5-layered composites were similar, although the 5-layered composites showed a slightly lower thermal diffusivity due to the presence of the additional boundaries and a higher number of interfaces between graphene nanoplatelets and SiC matrix.
4. The electrical conductivity increased in all composites with increasing content of GNPs in particular SiC layer. The highest electrical conductivity (1442 S/m) was observed in the SiC layer with 15 wt.% of GNPs (S15GNP) in 3-layered composites sintered under 50 MPa pressure. The electrical conductivity decreased to 869 S/m in the 5-layered composite due to the presence of layers with lower electrical conductivities (SiC and S5GNP layers) between the S15GNP surface layers. However, the main advantage of 5-layered composites is the possibility of their effective assisting electrode EDM machining from both sides, which is not possible for the 3-layered composites from the SiC side (σ = 122 S/m), only from the side containing 15 wt.% GNPs.
5. The investigation of mechanical properties showed a stepwise decrease of instrumented hardness and elastic modulus in the individual layers with increasing GNPs content. The coefficient of friction increased from 0.38 for the SiC layer to 0.62 for the layer with 15 wt.% GNPs, and no lubrication effect of graphene platelets was observed. The changes of mechanical properties on the interfaces were remarkable, as the individual layers were well bonded to each other with sharp, well-defined interfaces.

## Figures and Tables

**Table 1:** Properties of all layered materials and reference, non-layered materials.

Sample	Type of Samples	Thickness of Layers (μm)	RD (%)	Thermal Diffusivity (mm^2^/s)	Electrical Conductivity (S/m)
C3-7	3-layered	SiC: 1200–1300	92.2	22.2	588.2 ± 8.5
C3-30	3-layered	S5GNP: 900–1000	98.1	29.2	1310.1 ± 36.5
C3-40	3-layered	S15GNP: 1300–1400	98.6	28.6	1437.9 ± 73.1
C3-50	3-layered	S15GNP: 1100–1200	98.8	26.2	1441.8 ± 59.4
C5-7	5-layered	S5GNP: 900–1000	95.4	27.4	523.4 ± 27.0
C5-30	5-layered	SiC: 1300–1400	97.5	24.7	637.3 ± 32.5
C5-40	5-layered	S5GNP: 900–1000	98.8	27.0	740.1 ± 40.5
C5-50	5-layered	S15GNP: 1100–1200	98.7	25.8	843.5 ± 34.6
SiC ref. [2]	Non-layered		99.5	37.4	100.3 ± 2.0
SiC + 5% GNP [2]	Non-layered		98.5	33.8	528.5 ± 66.8
SiC + 15% GNPs [2]	Non-layered		97.6	23.9	2031.3 ± 22.9

## Data Availability

Data sharing is not applicable.

## References

[1] König W., Dauw D., Levy G., Panten U. (1988). EDM-Future Steps towards the Machining of Ceramics. CIRP Ann..

[2] Hanzel O., Singh M.A., Marla D., Sedlák R., Šajgalík P. (2019). Wire electrical discharge machinable SiC with GNPs and GO as the electrically conducting filler. J. Eur. Ceram. Soc..

[3] Singh M.A., Sarma D.K., Hanzel O., Sedláček J., Šajgalík P. (2017). Machinability analysis of multi walled carbon nanotubes filled alumina composites in wire electrical discharge machining process. J. Eur. Ceram. Soc..

[4] Mohri N., Fukuzawa Y., Tani T., Saito N., Furutani K. (1996). Assisting Electrode Method for Machining Insulating Ceramics. CIRP Ann..

[5] Rajurkar K., Sundaram M., Malshe A. (2013). Review of Electrochemical and Electrodischarge Machining. Procedia CIRP.

[6] Hanzel O., Lenčéš Z., Kim Y.-W., Fedor J., Šajgalík P. (2020). Highly electrically and thermally conductive silicon carbide-graphene composites with yttria and scandia additives. J. Eur. Ceram. Soc..

[7] Sedlák R., Kovalčíková A., Múdra E., Rutkowski P., Dubiel A., Girman V., Bystrický R., Dusza J. (2017). Boron carbide/graphene platelet ceramics with improved fracture toughness and electrical conductivity. J. Eur. Ceram. Soc..

[8] Ramirez C., Figueiredo F.M., Miranzo P., Poza P., Osendi M.I. (2012). Graphene nanoplatelet/silicon nitride composites with high electrical conductivity. Carbon.

[9] Centeno A., Rocha V., Alonso B., Fernández A., Gutierrez-Gonzalez C., Torrecillas R., Zurutuza A. (2013). Graphene for tough and electroconductive alumina ceramics. J. Eur. Ceram. Soc..

[10] Miranzo P., Ramirez C., Román-Manso B., Garzón L., Gutiérrez H.R., Terrones M., Ocal C., Osendi M.I., Belmonte M. (2013). In situ processing of electrically conducting graphene/SiC nanocomposites. J. Eur. Ceram. Soc..

[11] Román-Manso B., Figueiredo F.M., Achiaga B., Barea R., Pérez-Coll D., Morelos-Gómez A., Terrones M., Osendi M.I., Belmonte M., Miranzo P. (2016). Electrically functional 3D-architectured graphene/SiC composites. Carbon.

[12] Tan Y., Zhang H., Peng S. (2016). Electrically conductive graphene nanoplatelet/boron carbide composites with high hardness and toughness. Scr. Mater..

[13] Román-Manso B., Chevillotte Y., Osendi M.I., Belmonte M., Miranzo P. (2016). Thermal conductivity of silicon carbide composites with highly oriented graphene nanoplatelets. J. Eur. Ceram. Soc..

[14] Tan Y., Luo H., Zhang H., Peng S. (2016). Graphene nanoplatelet reinforced boron carbide composites with high electrical and thermal conductivity. J. Eur. Ceram. Soc..

[15] Celik Y., Çelik A., Flahaut E., Suvaci E. (2016). Anisotropic mechanical and functional properties of graphene-based alumina matrix nanocomposites. J. Eur. Ceram. Soc..

[16] Kultayeva S., Ha J.-H., Malik R., Kim Y.-W., Kim K.J. (2020). Effects of porosity on electrical and thermal conductivities of porous SiC ceramics. J. Eur. Ceram. Soc..

[17] Cho T.-Y., Kim Y.-W., Kim K.J. (2016). Thermal, electrical, and mechanical properties of pressureless sintered silicon carbide ceramics with yttria-scandia-aluminum nitride. J. Eur. Ceram. Soc..

[18] Kim Y.-W., Cho T.-Y., Kim K.J. (2015). Effect of grain growth on electrical properties of silicon carbide ceramics sintered with gadolinia and yttria. J. Eur. Ceram. Soc..

[19] Cho T.-Y., Kim Y.-W. (2017). Effect of grain growth on the thermal conductivity of liquid-phase sintered silicon carbide ceramics. J. Eur. Ceram. Soc..

[20] Jang S.H., Kim Y.-W., Kim K.J., Lee S.-J., Lim K.-Y. (2016). Effects of Y2 O3 -RE2 O3 (RE = Sm, Gd, Lu) Additives on Electrical and Thermal Properties of Silicon Carbide Ceramics. J. Am. Ceram. Soc..

[21] Zhou Y., Hirao K., Yamauchi Y., Kanzaki S. (2003). Effects of rare-earth oxide and alumina additives on thermal conductivity of liquid-phase-sintered silicon carbide. J. Mater. Res..

[22] Yu M., Picot O.T., Saunders T.G., Dlouhý I., Feng J., Titirici M.-M., Mahajan A., Reece M.J. (2018). Graphene-reinforced silicon oxycarbide composites prepared by phase transfer. Carbon.

[23] Llorente J., Román-Manso B., Miranzo P., Belmonte M. (2016). Tribological performance under dry sliding conditions of graphene/silicon carbide composites. J. Eur. Ceram. Soc..

[24] Xia H., Zhang X., Shi Z., Zhao C., Li Y., Wang J., Qiao G. (2015). Mechanical and thermal properties of reduced graphene oxide reinforced aluminum nitride ceramic composites. Mater. Sci. Eng. A.

[25] Kovalčíková A., Tatarko P., Sedlák R., Medveď D., Chlup Z., Múdra E., Dusza J. (2020). Mechanical and tribological properties of TiB2-SiC and TiB2-SiC-GNPs ceramic composites. J. Eur. Ceram. Soc..

[26] Sedlák R., Kovalčíková A., Balko J., Rutkowski P., Dubiel A., Zientara D., Girman V., Múdra E., Dusza J. (2017). Effect of graphene platelets on tribological properties of boron carbide ceramic composites. Int. J. Refract. Met. Hard Mater..

[27] Liu X., Li J., Yu X., Fan H., Wang Q., Yan S., Wang L., Jiang W. (2016). Graphene nanosheet/titanium carbide composites of a fine-grained structure and improved mechanical properties. Ceram. Int..

[28] Clegg W., Kendall K., Alford N.M., Button T.W., Birchall J.D. (1990). A simple way to make tough ceramics. Nat. Cell Biol..

[29] Belmonte M., Nistal A., Cruz-Silva R., Morelos-Gómez A., Terrones M., Miranzo P., Osendi M.I. (2015). Directional Electrical Transport in Tough Multifunctional Layered Ceramic/Graphene Composites. Adv. Electron. Mater..

[30] An Y., Han J., Zhang X., Han W., Cheng Y., Hu P., Zhao G. (2016). Bioinspired high toughness graphene/ZrB2 hybrid composites with hierarchical architectures spanning several length scales. Carbon.

[31] Rincón A., Moreno R., Gutiérrez-González C.F., Sainz R., Salvador M.D., Borrell A. (2016). Colloidal processing of fully stabilized zirconia laminates comprising graphene oxide-enriched layers. J. Eur. Ceram. Soc..

[32] Balázsi K., Furkó M., Liao Z., Gluch J., Medved D., Sedlák R., Dusza J., Zschech E. (2020). Porous sandwich ceramic of layered silicon nitride-zirconia composite with various multilayered graphene content. J. Alloy. Compd..

[33] Balázsi K., Furkó M., Liao Z., Fogarassy Z., Medved D., Zschech E., Dusza J. (2020). Graphene added multilayer ceramic sandwich (GMCS) composites: Structure, preparation and properties. J. Eur. Ceram. Soc..

[34] Hong C.-Q., Zhang X.-H., Li W.-J., Han J.-C., Meng S.-H. (2008). A novel functionally graded material in the ZrB2–SiC and ZrO2 system by spark plasma sintering. Mater. Sci. Eng. A.

[35] Nakashima S., Harima H. (1997). Raman investigation of SiC polytypes. Phys. Stat. Sol..

[36] Rohbeck N., Xiao P. (2014). Effects of thermal treatment on the mechanical integrity of silicon carbide in HTR fuel up to 2200 °C. J. Nucl. Mater..

[37] Zhu K., Guo L., Lin J., Hao W., Shang J., Jia Y., Chen L., Jin S., Wang W., Chen X. (2012). Graphene covered SiC powder as advanced photocatalytic material. Appl. Phys. Lett..

[38] Miranzo P., López-Mir L., Román-Manso B., Belmonte M., Osendi M.I., Ocal C. (2016). Prominent local transport in silicon carbide composites containing in-situ synthesized three-dimensional graphene networks. J. Eur. Ceram. Soc..

[39] Ferrari A.C., Meyer J.C., Scardaci V., Casiraghi C., Lazzeri M., Mauri F., Piscanec S., Jiang D., Novoselov K.S., Roth S. (2006). Raman Spectrum of Graphene and Graphene Layers. Phys. Rev. Lett..

[40] Barros E.B., Demir N.S., Filho A.G.S., Filho J.M., Jorio A., Dresselhaus G., Dresselhaus M.S. (2005). Raman spectroscopy of graphitic foams. Phys. Rev. B.

[41] Hanzel O., Sedlák R., Sedláček J., Bizovská V., Bystrický R., Girman V., Kovalčíková A., Dusza J., Šajgalík P. (2017). Anisotropy of functional properties of SiC composites with GNPs, GO and in-situ formed graphene. J. Eur. Ceram. Soc..

[42] Sedlák R., Kovalčíková A., Girman V., Múdra E., Rutkowski P., Dubiel A., Dusza J. (2017). Fracture characteristics of SiC/graphene platelet composites. J. Eur. Ceram. Soc..

[43] Ramirez C., Miranzo P., Belmonte M., Osendi M.I., Poza P., Vega S., Terrones M. (2014). Extraordinary toughening enhancement and flexural strength in Si3N4 composites using graphene sheets. J. Eur. Ceram. Soc..

